# Redox control at the ER-mitochondria interface in kidney transplantation: MAM-centered stress signaling and translational organoid platforms

**DOI:** 10.1016/j.redox.2026.104236

**Published:** 2026-05-26

**Authors:** Baicheng Kuang, Lin Han, Sopheaktra Tan, Sokun Tan, Sopheap Bou, Yuanyuan Zhao, Yan Li, Jiasheng Yu, Nianqiao Gong

**Affiliations:** a Institute of Organ Transplantation, Tongji Hospital, Tongji Medical College, Huazhong University of Science and Technology, Key Laboratory of Organ Transplantation, Ministry of Education, Chinese Academy of Medical Sciences, NHC Key Laboratory of Organ Transplantation, Wuhan, 430030, China; b Tongji Junshan Neuroscience Center, Tongji Hospital, Tongji Medical College, Huazhong University of Science and Technology, Wuhan, 430030, China; c Department of Neurosurgery, Tongji Hospital, Tongji Medical College, Huazhong University of Science and Technology, Wuhan, 430030, China; d Nephrology, KCU and Hemodialysis Department, Cambodia-China Friendship Preah Kossamak Hospital, Phnom Penh, 12000, Cambodia; e Anesthesia and Reanimation, Emergency, ICU Department, Cambodia-China Friendship Preah Kossamak Hospital, Phnom Penh, 12000, Cambodia; f Urology Department, Cambodia-China Friendship Preah Kossamak Hospital, Phnom Penh, 12000, Cambodia; g Key Laboratory of Animal Biological Products & Genetic Engineering, Ministry of Agriculture and Rural, Sinopharm Animal Health Corporation Ltd, Wuhan, 430030, China

**Keywords:** Oxidative stress, Endoplasmic reticulum stress, Mitochondria-associated membranes, Mitochondrial dysfunction, Kidney transplantation, Organoid

## Abstract

Kidney transplantation is inevitably accompanied by ischemia-reperfusion injury in which oxidative stress and endoplasmic reticulum (ER) stress act as tightly interconnected drivers of mitochondrial dysfunction, inflammation, and long-term graft failure. Excessive reactive oxygen species disrupt mitochondrial homeostasis, while unresolved ER stress activates maladaptive unfolded protein response signaling, together shaping tubular cell fate. Although these processes have been extensively studied, their spatial and functional integration remains incompletely understood. Growing evidence indicates that oxidative stress and ER stress converge at mitochondria-associated membranes (MAMs), where calcium signaling, redox regulation, and stress-adaptive networks are integrated. However, the dynamic and context-dependent nature of MAM remodeling remains poorly defined and difficult to investigate using conventional experimental systems. In this review, we propose a MAM-centered framework that integrates cellular stress responses, with a particular focus on ischemia-reperfusion in kidney transplantation. We further highlight therapeutic strategies targeting MAM-associated pathways, including mitochondria-directed antioxidants, ER oxidoreductases and structural and signaling proteins of MAM. In parallel, we summarize emerging kidney organoid platforms as human-relevant translational systems for modeling MAM dynamics under controlled conditions. By integrating mechanistic insights with organoid-based investigations, this review bridges a critical gap between molecular understanding and translational application, and offers a conceptual framework for MAM-targeted strategies aimed at improving graft resilience and long-term transplant outcomes.

## Highlights

• MAM-centered integrative framework links redox stress, ER stress, and mitochondrial dysfunction in kidney transplantation.
• Self-amplifying oxidative-inflammatory circuits underlie ischemia-reperfusion injury and graft vulnerability.
• MAM-associated signaling nodes serve as therapeutic targets for restoring mitochondrial quality control and cellular homeostasis.
• Kidney organoids as emerging platforms bridge mechanistic discovery and translational intervention in transplant research.

## Graphical abstract

**Figure:**
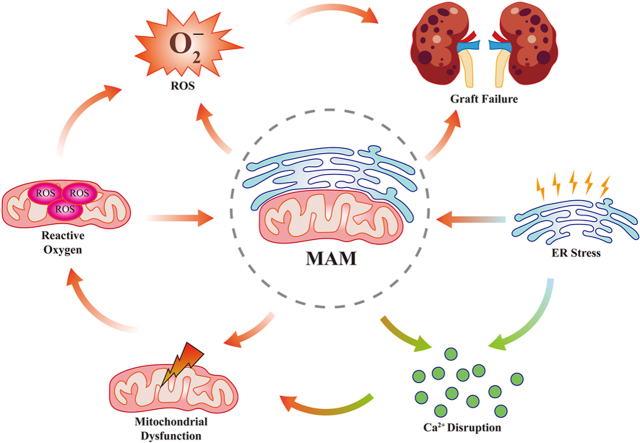

## 1 Introduction

Kidney transplantation remains the most effective therapy for end-stage renal disease; however, ischemia-reperfusion injury (IRI) continues to compromise early graft function and long-term survival [1]. A central feature of renal IRI is the profound disturbance of cellular redox homeostasis, characterized by excessive generation of reactive oxygen species (ROS), mitochondrial dysfunction, and activation of stress-responsive signaling pathways [2]. Although these processes have been extensively studied and are known to converge in driving tubular injury, inflammation, and maladaptive repair, ultimately predisposing grafts to chronic dysfunction, they are largely examined as parallel pathways. A unifying framework that explains how they are spatially and functionally integrated during transplantation remains lacking.

Accumulating evidence from our group and other researchers indicate that oxidative stress is closely associated with endoplasmic reticulum (ER) stress during transplantation [[3], [4], [5]]. Perturbation of protein folding capacity activates the unfolded protein response (UPR), a conserved adaptive program mediated through the IRE1-XBP1, PERK-eIF2α-ATF4, and ATF6 pathways. Although transient UPR activation supports cellular adaptation, sustained or dysregulated ER stress amplifies oxidative injury, disrupts calcium homeostasis, and promotes apoptosis and fibrosis [6]. Notably, mitochondria-associated membranes (MAMs) have emerged as critical signaling hubs coordinating redox signaling, Ca^2+^ transfer, and metabolic adaptation between the ER and mitochondria [7,8]. MAM finely tune ER-mitochondrial coupling and Ca^2+^ transfer, thereby shaping mitochondrial bioenergetics and cellular stress resilience during IRI and perturbed ER redox homeostasis. While previous reviews have primarily treated MAMs as structural or biochemical entities, or examined oxidative stress, ER stress, and mitochondrial dysfunction, the present manuscript provides a unified and integrative framework that positions MAMs as dynamic signaling interfaces coordinating stress-responsive inter-organelle communication in kidney transplantation.

Despite major advances in delineating these pathways in experimental models, mechanistic investigation in human kidney transplantation remains limited. Animal models incompletely recapitulate human-specific redox regulation, aging-related vulnerability, and donor heterogeneity, whereas clinical biopsies offer only static snapshots of highly dynamic processes. These constraints have hindered precise dissection of oxidative stress-ER stress crosstalk and have limited translation to mechanistically-informed therapies. Recent progress in human kidney organoid technology provides new opportunities to bridge this gap [9]. Derived from pluripotent or adult renal progenitors, kidney organoids recapitulate key nephron architectures and enable controlled modeling of hypoxia-reoxygenation events, inflammatory stress, metabolic perturbations, and genetic susceptibility in a human-relevant context [10,11]. When combined with advanced tools such as reporter systems, CRISPR-based editing, perfusion platforms, and multi-omics profiling, organoids offer a powerful experimental framework to interrogate redox biology, ER stress signaling, and organelle crosstalk during transplantation [[12], [13], [14]]. Importantly, many defining features of MAM biology, including dynamic ER-mitochondria tethering, localized calcium microdomains, and redox-sensitive remodeling, are highly context- and cell-state-dependent processes, making them difficult to resolve using conventional static biopsy samples or reductionist in vitro systems. In this regard, kidney organoids provide an experimentally tractable, human-relevant platform for interrogating dynamic MAM remodeling under controlled ischemic and inflammatory conditions.

In this review, we summarize recent advances in delineating the integrated molecular networks underlying oxidative stress- and ER stress-driven cellular injury in kidney transplantation, with a particular focus on MAM as central signaling hubs. In parallel, we highlight kidney organoids as next-generation platforms for modeling these complex stress responses in human-relevant settings, thereby bridging mechanistic insight with the translational development of MAM-targeted precision therapies.

## 2 Oxidative stress in kidney transplantation

Oxidative stress represents a central pathogenic driver of kidney transplant injury, particularly during IRI, when abrupt fluctuations in oxygen availability profoundly disrupt redox homeostasis [15]. Renal tubular epithelial cells, characterized by high metabolic demand and mitochondrial density, are especially vulnerable to the effects of reactive oxygen species (ROS) [16]. Excessive ROS generation not only causes direct macromolecular damage but also initiates signaling cascades that amplify inflammation, cell death, and maladaptive repair, ultimately contributing to chronic allograft dysfunction [17].

### 2.1 Sources of ROS during renal ischemia-reperfusion

Multiple cellular and enzymatic sources contribute to ROS overproduction during kidney transplantation. Mitochondria represent the predominant source, particularly at complexes I and III of the electron transport chain, where electron leakage is markedly enhanced during ischemia and early reperfusion [15,18]. Ischemic adenosine triphosphate (ATP) depletion leads to mitochondrial depolarization, accumulation of reduced electron carriers, and succinate buildup, which, upon reperfusion, drives reverse electron transport and a burst of superoxide generation [18]. In addition to mitochondria, several enzymatic systems contribute to oxidative stress in transplanted kidneys. NADPH oxidases (NOX family), especially NOX2 and NOX4, are activated in tubular epithelial cells, endothelial cells, and infiltrating immune cells, generating sustained ROS signals that promote inflammation and fibrosis [19]. Xanthine oxidoreductase (XOR) activation during ischemia further augments superoxide production, while uncoupled nitric oxide synthase (NOS) contributes to reactive nitrogen species formation [19,20]. Inflammatory cell infiltration, involving neutrophils and monocyte/macrophage, following reperfusion amplifies oxidative burden through respiratory burst activity, creating a self-reinforcing oxidative microenvironment.

Collectively, these ROS-generating systems do not function independently but instead form a highly interconnected oxidative network. Mitochondrial electron leakage, enzymatic ROS production, and inflammation-associated oxidative bursts dynamically reinforce one another, driving sustained redox imbalance, metabolic dysfunction, inflammatory activation, and tubular injury. Importantly, while moderate ROS signaling may initially support adaptive stress responses and cellular homeostasis, excessive or persistent ROS accumulation promotes oxidative damage, lipid peroxidation, mitochondrial dysfunction, and progressive graft injury.

### 2.2 Mitochondrial dysfunction as a central amplifier of oxidative injury

Mitochondrial impairment lies at the core of oxidative stress-mediated renal injury [21]. Under ischemic conditions, mitochondrial membrane potential (ΔΨm) is markedly reduced and ATP stores become exhausted. This is accompanied by acidosis, secondary to lactate accumulation and an increase in the intracellular calcium (Ca^2+^) concentration [22,23]. The outer mitochondrial membrane (OMM) remains intact and the mitochondrial permeability transition pore (mPTP) remains closed [24]. With subsequent reperfusion, the reintroduction of oxygen leads to a rapid normalization of pH and a rapid restoration of ΔΨm, precipitating a range of adverse sequelae including the production of mitochondrial ROS, exacerbation of Ca^2+^ overload, OMM destruction, and mPTP formation [25]. Finally, with the opening of the mPTP, the inner membrane loses selectivity for small molecules, protons flow freely, and ΔΨm undergoes irreversible and complete collapse [26]. Most of the oxidative damage can be attributed to such mitochondrial dysfunction. In addition, during IRI, mitochondrial dynamics are profoundly altered, with excessive fission, cristae disruption, and impaired mitophagy leading to accumulation of dysfunctional organelles. These damaged mitochondria exhibit reduced oxidative phosphorylation capacity and exaggerated ROS leakage, perpetuating redox imbalance.

Mitochondrial DNA (mtDNA), which lacks protective histones and robust repair mechanisms, is particularly susceptible to oxidative damage [27]. Release of oxidized mtDNA into the cytosol or extracellular space activates innate immune pathways, including Cyclic GMP-AMP Synthase - Stimulator of Interferon Genes (cGAS-STING) and Toll-like receptor (TLR) signaling, thereby linking mitochondrial injury to sterile inflammation [28]. These inflammatory signals attract and activate immune cells such as macrophages and neutrophils, and promote more ROS generation [28,29]. In parallel, mPTP opening contributes to loss of membrane potential, ATP depletion, and necrotic or apoptotic cell death. Together, these events establish mitochondria not merely as passive victims but as active amplifiers of oxidative injury during transplantation.

### 2.3 Downstream molecular consequences of oxidative stress

Excessive ROS accumulation exerts wide-ranging effects on cellular macromolecules and signaling networks [30]. Lipid peroxidation alters membrane integrity and generates reactive aldehydes such as 4-hydroxynonenal, which further propagate oxidative damage and modify protein function [31]. Oxidative modification of proteins disrupts enzymatic activity, impairs folding and increases proteotoxic stress, thereby intersecting with endoplasmic reticulum (ER) stress pathways [32,33]. At the signaling level, ROS activate multiple redox-sensitive pathways, including MAPKs, NF-κB, and inflammasome components, promoting the transcription of pro-inflammatory cytokines and chemokines [32]. Sustained oxidative stress also influences cell fate decisions by modulating BCL-2 family proteins, caspase activation, and regulated necrosis pathways [30]. Importantly, oxidative injury interferes with tubular cell cycle progression and differentiation, contributing to maladaptive repair and fibrotic remodeling rather than functional regeneration [34].

**Fig. 1:**
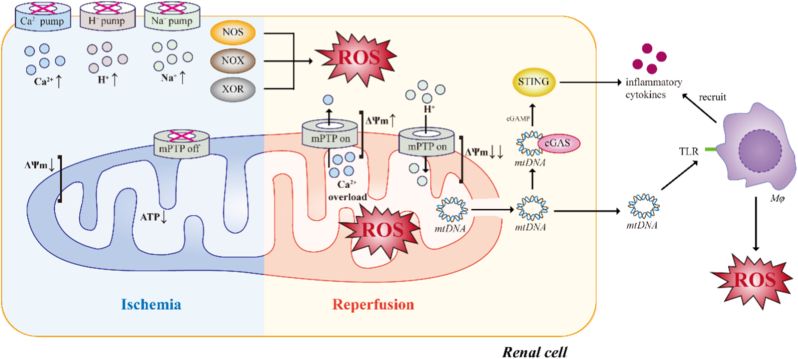
Schematic illustration of mitochondrial dysfunction-driven oxidative and inflammatory amplification during ischemia-reperfusion injury. Schematic representation of mitochondrial dysfunction–driven oxidative and inflammatory amplification during renal ischemia–reperfusion injury (IRI). During ischemia, oxygen deprivation suppresses mitochondrial oxidative phosphorylation, resulting in ATP depletion, dissipation of mitochondrial membrane potential (ΔΨm), ionic imbalance, and intracellular Ca^2+^ accumulation. Upon reperfusion, rapid reoxygenation triggers excessive mitochondrial ROS generation, Ca^2+^ overload, and opening of the mitochondrial permeability transition pore (mPTP), leading to further ΔΨm collapse, mitochondrial dysfunction, and release of mitochondrial DNA (mtDNA). Cytosolic mtDNA activates innate immune pathways, including cGAS-STING and Toll-like receptor (TLR) signaling, promoting inflammatory cytokine production and recruitment of immune cells, particularly macrophages. Activated immune cells further amplify oxidative stress through respiratory burst activity, establishing a self-sustaining oxidative–inflammatory feed-forward loop that drives tubular epithelial injury and graft dysfunction.

Collectively, these processes position oxidative stress as a unifying upstream driver that links mitochondrial dysfunction, inflammation, cell death, and chronic structural remodeling in kidney transplantation (Fig. 1). However, oxidative stress rarely acts in isolation. Instead, it is tightly integrated with other stress response systems - most notably ER stress - through coordinated signaling networks that determine cellular adaptation or failure. Understanding this integration is essential for identifying effective therapeutic targets, as discussed in the following sections.

## 3 ER stress and UPR signaling in kidney transplantation

ER stress represents a fundamental cellular response to disturbances in protein folding homeostasis and is increasingly recognized as a central contributor to kidney transplant injury. During ischemia-reperfusion, abrupt fluctuations in oxygen and nutrient supply, ATP depletion, oxidative imbalance, and calcium dysregulation collectively overwhelm the protein-folding capacity of the ER, leading to the accumulation of misfolded or unfolded proteins [35,36]. To restore proteostasis, cells activate the UPR, an evolutionarily conserved signaling network coordinated by three principal ER stress sensors: inositol-requiring enzyme 1 (IRE1), protein kinase RNA-like ER kinase (PERK), and activating transcription factor 6 (ATF6) [[37], [38], [39]]. While transient UPR activation serves adaptive and cytoprotective functions, persistent or excessive ER stress promotes inflammation, mitochondrial dysfunction, and programmed cell death, thereby contributing to graft injury and impaired recovery after transplantation.

### 3.1 Canonical UPR pathways in kidney injury

#### 3.1.1 IRE1-XBP1 signaling

Of the three UPR pathways, the IRE1-XBP1 axis is the most evolutionarily conserved and plays a pivotal role in maintaining ER homeostasis. Upon ER stress, IRE1 undergoes oligomerization and autophosphorylation, activating its endoribonuclease (RNase) activity to splice X-box binding protein 1 (XBP1) mRNA. The spliced isoform, XBP1s, subsequently functions as a transcription factor that promotes expression of genes involved in protein folding, ER-associated degradation (ERAD), lipid biosynthesis, and redox balance [22,40]. Accumulating evidence indicates that the IRE1-XBP1 axis plays a critical role in tubular epithelial cell survival under stress conditions. Experimental studies demonstrate rapid activation of IRE1-XBP1 signaling following ischemic insult, contributing to adaptive responses that restore proteostasis [41]. In this setting, transient XBP1 activation appears to function as a protective mechanism that limits proteotoxic injury and supports tubular survival during early reperfusion. However, the role of XBP1 signaling in kidney injury is highly context-dependent and time-dependent. For example, downregulation of XBP1 has been shown to accelerate tubular injury and fibrotic remodeling following acute kidney injury, suggesting that impaired adaptive UPR signaling compromises long-term repair capacity [42]. Beyond its canonical role in protein quality control, XBP1 also regulates mitochondrial metabolism, antioxidant capacity, and cellular bioenergetics, thereby linking ER stress to mitochondrial function [43,44]. In addition to the RNase activities, IRE1 also possesses kinase activities. Dysregulation of IRE1 signaling can shift its output toward pro-death pathways through regulated IRE1-dependent decay (RIDD) of mRNAs and activation of stress kinases. Notably, allosteric inhibitors such as KIRA6 attenuate IRE1 RNase activity and have been shown to reduce ER stress-induced cell death and inflammation in preclinical models [45]. Taken together, these findings suggest that the biological consequences of IRE1–XBP1 signaling during renal IRI are not uniformly protective or deleterious, but instead depend on the magnitude, duration, and cellular context of ER stress activation.

#### 3.1.2 PERK-eIF2α-ATF4 pathway

The PERK pathway constitutes another major arm of the UPR activated during renal stress. Following ER stress, PERK phosphorylates eukaryotic initiation factor 2α (eIF2α), resulting in transient global translational attenuation that reduces the protein-folding burden [46]. At the same time, selective translation of activating transcription factor 4 (ATF4) is induced, driving expression of genes involved in amino acid metabolism, redox regulation, and autophagy [47,48]. In the context of kidney transplantation, PERK-eIF2α signaling has a dual role [49]. Early activation may protect tubular cells by limiting protein overload and enhancing adaptive stress responses [50]. However, sustained activation promotes the expression of C/EBP homologous protein (CHOP), a key mediator of apoptosis [51]. Elevated CHOP expression has been consistently associated with tubular cell death, inflammation, and worse functional recovery following ischemic injury [52]. Thus, the PERK pathway represents a critical switch between adaptive and maladaptive ER stress responses in renal transplantation.

#### 3.1.3 ATF6 signaling

ATF6 serves as the third major sensor of ER stress and operates through a distinct activation mechanism. Under stress conditions, ATF6 translocates from the ER to the Golgi apparatus, where it undergoes proteolytic cleavage to release an active transcription factor that upregulates ER chaperones and components of the ERAD machinery [36]. Compared with IRE1 and PERK, ATF6 signaling is generally considered cytoprotective, enhancing protein-folding capacity and facilitating recovery from transient stress. In kidney injury models, ATF6 activation has been associated with improved cellular resilience and reduced apoptosis [53]. However, its precise contribution during renal transplantation remains incompletely defined [54]. Emerging evidence suggests that insufficient or dysregulated ATF6 activation may compromise adaptive proteostasis and predispose tubular cells to persistent ER stress, particularly under conditions of prolonged ischemia or oxidative overload [54,55].

### 3.2 Adaptive versus maladaptive ER stress responses

A defining feature of ER stress signaling in kidney transplantation is its dynamic transition from adaptive to maladaptive responses. During early or moderate stress, coordinated activation of UPR pathways promotes restoration of ER homeostasis through enhanced folding capacity, degradation of misfolded proteins, metabolic rewiring, and temporary suppression of protein synthesis [56]. This adaptive phase supports tubular cell survival and facilitates functional recovery after reperfusion. However, when ER stress is severe or prolonged, as often occurs in marginal donor kidneys, extended cold ischemia, or settings of heightened oxidative burden, the UPR becomes maladaptive [57]. Persistent activation of PERK-CHOP signaling, sustained IRE1 activity, and impaired resolution of stress responses drive apoptosis, necroptosis, and inflammatory signaling [58]. These maladaptive processes contribute to tubular atrophy, interstitial inflammation, and fibrogenic remodeling, ultimately predisposing grafts to delayed graft function and chronic dysfunction.

### 3.3 ER stress in ischemia-reperfusion and transplant pathology

**Fig. 2:**
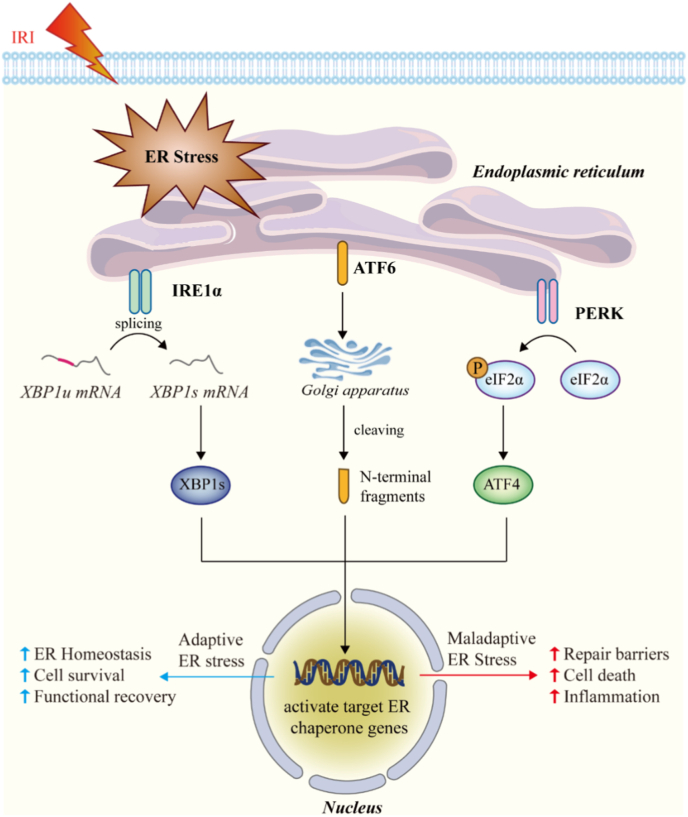
Integrated unfolded protein response signaling and adaptive-maladaptive ER stress transition during renal ischemia–reperfusion injury. Ischemia-reperfusion injury (IRI) triggers accumulation of misfolded proteins within the ER lumen, initiating the unfolded protein response (UPR) through activation of three canonical sensors: inositol-requiring enzyme 1α (IRE1α), activating transcription factor 6 (ATF6), and protein kinase RNA-like ER kinase (PERK). Activated IRE1 catalyzes unconventional splicing of X-box binding protein 1 (XBP1) mRNA, generating the transcriptionally active isoform XBP1s, which induces expression of ER chaperones and folding enzymes to restore proteostasis. ATF6 translocates to the Golgi apparatus for regulated intramembrane proteolysis, releasing its N-terminal fragment that enters the nucleus to upregulate adaptive UPR target genes. PERK phosphorylates eukaryotic initiation factor 2α (eIF2α), transiently attenuating global protein synthesis while selectively enhancing translation of ATF4, thereby promoting redox control, amino acid metabolism, and stress adaptation. Coordinated activation of these pathways supports ER homeostasis, cellular survival, and functional recovery during transient stress. However, sustained or excessive UPR activation drives a maladaptive ER stress program characterized by impaired repair capacity, heightened inflammatory signaling, mitochondrial dysfunction, and apoptotic cell death, thereby exacerbating tubular injury and contributing to graft dysfunction. This dynamic balance between adaptive and maladaptive ER stress critically determines renal cell fate during ischemia-reperfusion.

Substantial experimental evidence supports a central role of ER stress in renal IRI and transplant damage [59,60]. In both IRI and transplantation models, markers of ER stress, including GRP78, phosphorylated PERK, spliced XBP1, and CHOP, are rapidly upregulated in tubular epithelial cells following ischemia and reperfusion, reflecting robust activation of UPR signaling pathways. Genetic or pharmacological targeting of excessive ER stress signaling has been shown to modulate injury severity, underscoring their functional relevance [61]. ER stress is tightly coupled to tubular epithelial apoptosis, inflammatory cytokine release, and impaired regenerative responses. Persistent ER stress activation drives bidirectional crosstalk with mitochondrial dysfunction, resulting in calcium dyshomeostasis, mitochondrial depolarization, and excessive ROS production. These processes amplify cellular injury and propagate pro-inflammatory stress signals to adjacent nephron segments, thereby exacerbating tissue damage. Beyond the acute phase, unresolved ER stress impairs cell cycle re-entry and differentiation in surviving tubular epithelial cells, promoting maladaptive repair and progressive tubulointerstitial fibrosis, key determinants of chronic allograft dysfunction [62] (Fig. 2).

Collectively, these findings position ER stress not as a secondary epiphenomenon of ischemic injury, but as a central hub integrating metabolic stress, redox imbalance, inflammation, and cell fate decisions in kidney transplantation. Defining how ER stress interfaces with mitochondrial and oxidative signaling pathways is therefore essential for the development of targeted therapies to preserve graft function and improve long-term transplant outcomes. This integrative perspective naturally leads to the emerging concept of MAM as critical platforms coordinating ER-mitochondria communication under stress conditions, which is discussed in the following section.

## 4 MAM as integrative hubs linking redox and ER stress

MAM are specialized subcellular domains formed by close physical apposition between the ER and mitochondria, typically maintained at a distance of 10-30 nm [63]. Rather than static structures, MAM represent dynamic signaling platforms that coordinate multiple aspects of cellular homeostasis, including calcium exchange, lipid trafficking, redox signaling, mitochondrial dynamics, and stress adaptation [64,65]. Growing evidence indicates that disruption of MAM integrity is a convergent pathological feature in conditions characterized by oxidative stress and ER dysfunction [66,67]. Under physiological conditions, MAM tethering complexes maintain tightly regulated ER-mitochondria communication. During IRI, however, oxidative stress and proteostatic disruption drive dynamic remodeling of MAM architecture. In the context of kidney transplantation, where ischemia-reperfusion imposes simultaneous metabolic and proteostatic challenges, MAM emerge as critical integrative nodes that links ER stress to mitochondrial injury and determines tubular cell fate.

### 4.1 Structural and functional architecture

**Fig. 3:**
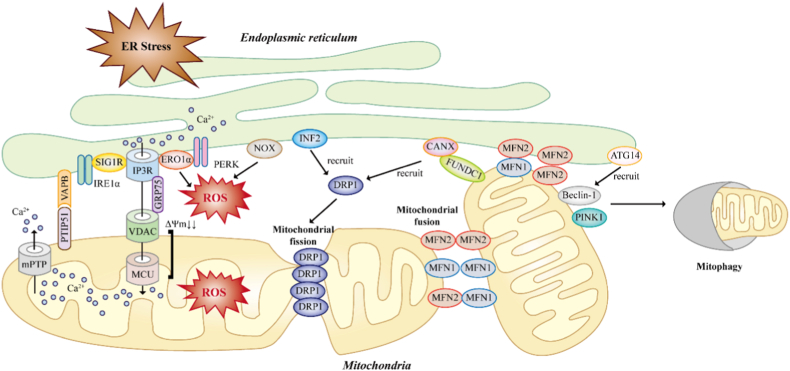
Mitochondria-associated membranes (MAMs) function as dynamic signaling hubs linking ER stress and oxidative stress during renal ischemia-reperfusion injury. Schematic illustration of the integrated signaling network centered at MAM that couples oxidative stress and endoplasmic reticulum (ER) stress to determine tubular cell survival or irreversible injury after kidney transplantation. Mitochondrial reactive oxygen species (ROS) generated during metabolic stress and reperfusion rapidly diffuse into the ER microenvironment, where they disrupt oxidative protein folding, impair disulfide bond formation, and destabilize luminal Ca^2+^ homeostasis, thereby activating the unfolded protein response (UPR) sensors. At MAM interfaces, dysregulated ER-to-mitochondria Ca^2+^ transfer, excessive ROS production, and altered redox signaling converge to drive mitochondrial depolarization, mitochondrial permeability transition pore (mPTP) opening, and Ca^2+^ release. Concurrent recruitment of INF2-DRP1 and FUNDC1-DRP1 promote pathological mitochondrial fission, while disruption of MFN1/2-mediated fusion and PINK1-dependent mitophagy impair mitochondrial quality control. Collectively, these interconnected signaling pathways define a MAM-centered regulatory hubs integrating ER stress, Ca^2+^ flux, mitochondrial dynamics, redox homeostasis, and mitophagy.

MAM are stabilized by a network of dynamic tethering proteins and signaling complexes that physically and functionally connect the ER and mitochondria. Among the best-characterized MAM-associated complexes is the inositol 1,4,5-trisphosphate receptors (IP3R)–glucose-regulated protein 75 (GRP75)–voltage-dependent anion channels (VDAC) axis, which facilitates efficient calcium transfer from the ER to mitochondria, thereby coupling ER calcium release to mitochondrial metabolism and bioenergetic adaptation [68,69]. Mitofusin-2 (MFN2), originally identified as a mitochondrial fusion protein, also contributes to ER-mitochondria tethering; however, its role in regulating contact site architecture remains context-dependent and incompletely defined. The molecular landscape of MAM regulation extends beyond these classical tethering complexes. The vesicle‐associated membrane protein‐associated protein B (VAPB)–protein tyrosine phosphatase‐interacting protein 51 (PTPIP51) complex functions as a structural bridge that promotes ER-mitochondria tethering and Ca^2+^ transfer [70]. Notably, PTPIP51, has been shown to exhibit phospholipid transfer activity, particularly for phosphatidic acid, linking MAM architecture to lipid trafficking and mitochondrial cardiolipin homeostasis. The Sigma-1 receptor (Sig-1R), a stress-responsive chaperone enriched at MAMs, stabilizes both IP3Rs and IRE1 at ER-mitochondria contact sites, thereby coordinating Ca^2+^ signaling and adaptive ER stress responses [71,72]. Consistent with these mechanistic insights, recent proteomic analyses demonstrate that MAM composition is dynamically remodeled under ischemic stress. Proteomic profiling of MAM fractions from hypoxia-reoxygenation-treated HK-2 cells identified 688 differentially expressed proteins, with MFN2 and BCL2 interacting protein 3 (BNIP3) emerging as key regulators of MAM integrity during renal IRI [73]. Additional regulatory proteins, including phosphofurin acidic cluster sorting protein-2 (PACS-2) and FUN14 domain-containing protein 1 (FUNDC1) further modulate MAM architecture and signaling efficiency [74] (Fig. 3).

Functionally, MAM serve as dynamic signaling interfaces integrating four tightly interconnected processes relevant to kidney injury, namely calcium signaling, lipid metabolism, redox regulation, and mitochondrial dynamics [75,76]. These processes do not operate independently but instead form an integrated stress-responsive network to maintain cellular homeostasis under physiological conditions and to shape adaptive responses during injury. Disruption of MAM integrity and signaling coordination promotes maladaptive remodeling and impaired cellular repair, ultimately contributing to graft injury and progressive dysfunction. Collectively, these findings indicate that MAMs function not merely as static structural contacts, but as dynamically regulated signaling platforms that integrate inter-organelle communication and coordinate stress adaptation in response to cellular injury.

### 4.2 Regulation of Ca^2+^ signaling and mitochondrial bioenergetics

Calcium transfer from the ER to mitochondria through MAM plays a pivotal role in regulating mitochondrial metabolism [77]. Physiological Ca^2+^ flux enhances the activity of key dehydrogenases in the tricarboxylic acid cycle, thereby supporting ATP production. However, excessive or dysregulated Ca^2+^ transfer - common during ER stress - results in mitochondrial Ca^2+^ overload, opening of the mPTP, collapse of membrane potential, and initiation of cell death pathways [78]. In renal tubular cells, ischemia-induced ATP depletion and ER calcium leakage synergize to amplify mitochondrial Ca^2+^ influx upon reperfusion [79]. This pathological Ca^2+^ transfer not only compromises mitochondrial respiration but also accelerates ROS generation, creating a vicious circle between calcium overload and oxidative stress. MAM integrity therefore critically determines whether calcium signaling remains adaptive or becomes cytotoxic during transplantation-related stress.

### 4.3 Regulators of redox homeostasis and ROS amplification

MAM represent important sites of redox regulation and ROS signaling. Several enzymes localized to or enriched at MAM participate directly in ROS production or sensing, including components of the electron transport chain, NADPH oxidases, and redox-sensitive chaperones [[80], [81], [82]]. Close ER-mitochondria proximity facilitates rapid propagation of redox signals, enabling ROS generated in mitochondria to influence ER function and vice versa. Under pathological conditions, excessive ROS disrupt MAM architecture and enhance aberrant ER-mitochondria coupling, which further promotes calcium overload and mitochondrial dysfunction [83]. Liu and colleagues demonstrated that GRP75 undergoes PKA-dependent phosphorylation and relocalization from mitochondria to MAMs and the cytosol under ferroptotic stress [84]. This spatial redistribution enables GRP75 to modulate NRF2–Keap1 signaling and suppress lipid peroxidation-driven cell death, thereby linking stress-responsive MAM remodeling to mitochondrial defense pathways. In renal transplantation, where ferroptosis and ROS bursts are key features of IRI, the PKA/GRP75 axis highlights the role of MAMs as dynamic signaling platforms that shape injury severity.

Emerging evidence further indicates that lipid peroxidation at MAMs directly alters the function of tethering complexes and converts these sites into pathological amplification nodes [85]. Reactive aldehydes, such as 4-hydroxynonenal (4-HNE), covalently modify cysteine residues on key MAM proteins, including GRP75, IP3R, and VDAC. Oxidative modification of GRP75 disrupts its bridging function within the IP3R–VDAC complex, leading to uncoupled ER-mitochondria Ca^2+^ transfer. Notably, MAM remodeling appears to be context-dependent. Under mild stress, dynamic adjustments in tethering and Ca^2+^ flux may support adaptive responses that sustain mitochondrial metabolism. By contrast, during sustained oxidative and ER stress, progressive lipid peroxidation and protein oxidation drive maladaptive reorganization of MAM contacts, amplifying Ca^2+^ dysregulation, ROS production, and mitochondrial dysfunction. This transition ultimately establishes a feed-forward loop in which oxidative stress and MAM dysfunction mutually reinforce one another and accelerate cellular injury.

### 4.4 MAM in mitochondrial dynamics and quality control

Beyond their established roles in calcium homeostasis and redox signaling, MAM also play a central role in regulating mitochondrial dynamics, including fission, fusion, and quality control. The outer mitochondrial membrane protein FUNDC1, recruited by the ER resident protein calnexin (CANX) accumulates at MAM in response to hypoxic or stress signals. It recruits dynamin-related protein 1 (DRP1) to drive mitochondrial fission prior to mitophagy. This process links mitochondrial fragmentation to clearance at ER-mitochondria contact sites [86,87]. Moreover, MAM concentrate multiple fission regulators including DRP1 receptors such as MFF, MiD49/51, and Fis1 at sites marked by ER tubules, enabling efficient mitochondrial division and subsequent autophagic removal of damaged organelles under stress conditions [88]. Inverted formin 2 (INF2), an ER-associated actin regulatory protein, further contributes to this process by promoting actin-dependent mitochondrial pre-constriction and facilitating DRP1 recruitment at MAM interfaces. Proper coordination of these events is essential for mitochondrial quality control and for cellular adaptation to stress. Under physiological conditions, transient mitochondrial fission and mitophagy facilitate the removal of damaged mitochondrial fragments, thereby preserving bioenergetic efficiency, and maintaining redox homeostasis. In renal IRI, excessive mitochondrial fission combined with impaired mitophagy leads to the accumulation of dysfunctional mitochondria and increased ROS production. Disruption of MAM-associated regulatory pathways exacerbates these defects, leading to persistence of damaged mitochondria and propagation of injury signals [24]. Thus, MAM function as spatial organizers that integrate mitochondrial dynamics with cellular stress responses.

### 4.5 MAM-centered integration of redox and ER stress

**Fig. 4:**
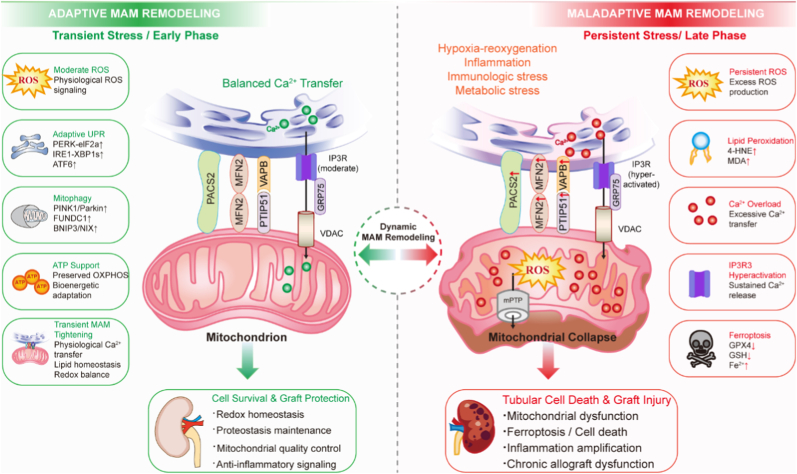
Adaptive-to-maladaptive MAM remodeling in kidney transplantation. Dynamic MAM remodeling during kidney transplantation-associated IRI. Under transient or early stress conditions (left panel), adaptive MAM remodeling promotes coordinated ER-mitochondria communication characterized by balanced Ca^2+^ transfer, moderate ROS signaling, adaptive UPR activation, mitophagy induction, and preservation of mitochondrial bioenergetics. Such responses facilitate controlled calcium exchange and mitochondrial quality control, thereby supporting redox homeostasis, proteostasis maintenance, and graft protection. In contrast, persistent hypoxia-reoxygenation, inflammation, metabolic stress, and oxidative overload drive maladaptive MAM remodeling (right panel). Excessive ROS accumulation, sustained IP3R activation, mitochondrial Ca^2+^ overload, lipid peroxidation, and mitochondrial permeability transition pore (mPTP) opening, collectively promote mitochondrial collapse, ferroptosis, inflammatory amplification, and graft injury. Hyperactivation of ER-mitochondria calcium transfer further exacerbates oxidative injury and mitochondrial dysfunction.

Oxidative stress and ER stress are tightly coupled at MAM and converge at the ER-mitochondria contact sites to form an integrated signaling network. Mitochondrial ROS generated during metabolic stress or ischemia-reperfusion can rapidly propagate to the ER microenvironment, where they impair oxidative protein folding, disrupt disulfide bond formation, and destabilize luminal calcium homeostasis [89]. These redox perturbations activate UPR sensors, including IRE1, PERK, and ATF6, initiating ER stress signaling. Conversely, unresolved ER stress feeds back to mitochondria by promoting ER Ca^2+^ release and excessive mitochondrial Ca^2+^ uptake at MAM interfaces, thereby stimulating electron transport chain activity, amplifying ROS production, and precipitating mitochondrial dysfunction [8]. Recent integrative studies further indicate that stress-induced remodeling of MAM architecture is itself a critical determinant of signaling outcomes [90]. Alterations in tethering protein composition, lipid microenvironment, and membrane fluidity modulate the efficiency of Ca^2+^ transfer and redox signal propagation. Under pathological conditions, oxidative damage and lipid peroxidation at MAM destabilize ER-mitochondria coupling, transforming physiological signaling microdomains into pathological amplification circuits. These structural changes reinforce a feed-forward loop in which redox stress, ER stress, and mitochondrial injury become mutually self-sustaining. Importantly, evidence positions MAM-associated signaling nodes, such as the IRE1-XBP1 axis, mitofusin-dependent tethering, and redox-sensitive chaperone systems, as critical regulators of this crosstalk [91]. Rather than functioning solely as downstream effectors, these pathways actively remodel MAM composition and function, thereby shaping calcium flux, mitochondrial bioenergetics, and cellular survival thresholds. In renal epithelial cells, which have high metabolic demand and strong mitochondrial dependence, disruption of this finely tuned MAM-centered integration renders cells particularly vulnerable to ischemic and inflammatory insults [69]. Collectively, MAM serve as both sensors and amplifiers of stress, translating localized redox and proteostatic disturbances into global decisions governing mitochondrial quality control, inflammation, and cell fate (Fig. 4). Conceptualizing kidney injury through this MAM-centric framework provides a unifying mechanistic basis for understanding how acute stress responses evolve into chronic dysfunction and highlights MAM-associated pathways as rational targets for therapeutic intervention.

## 5 Therapeutic strategies targeting MAM-associated pathways

As dynamic signaling interfaces integrating oxidative stress, ER stress, and mitochondrial dysfunction during IRI, MAM serve as attractive therapeutic targets for limiting transplant-associated injury. Modulation of MAM-associated pathways influences the balance between adaptive stress responses and maladaptive injury amplification, thereby regulating mitochondrial quality control, inflammatory signaling, ferroptosis susceptibility, and tubular cell survival. In this section, we summarize current mechanistically informed therapeutic strategies targeting MAM-associated signaling networks, while critically evaluating their experimental context, translational limitations, and potential relevance to kidney transplantation.

### 5.1 Mitochondria-directed antioxidants

A growing body of evidence supports the therapeutic potential of mitochondria-targeted antioxidants in attenuating oxidative injury associated with renal ischemia-reperfusion and transplantation-related stress. Given the central role of mitochondrial ROS (mtROS) bursts, ATP depletion, and bioenergetic failure of renal IRI, mitochondria-targeted interventions have attracted increasing attention as strategies to mitigate tubular injury and preserve graft function. However, although these approaches are mechanistically relevant to MAM-associated stress signaling, direct evidence demonstrating restoration of MAM integrity or ER-mitochondria communication remains limited. Among the most extensively studied agents, the lipophilic cation-conjugated antioxidant MitoQ has shown protective effects in experimental models of renal injury. Hamed et al. demonstrated that MitoQ significantly ameliorated renal IRI in transplantation settings by suppressing mitochondrial oxidative stress and preserving mitochondrial integrity [92]. Similarly, Xiao et al. reported that MitoQ attenuated tubular injury in diabetic kidney disease through activation of the NRF2–PINK1 pathway, thereby promoting mitophagy and limiting mtROS accumulation [93]. However, neither study directly assessed MAM architecture, ER-mitochondria contact integrity, or MAM-dependent calcium signaling. Accordingly, any proposed relationship between MitoQ-mediated protection and MAM stabilization should be considered mechanistically inferred rather than experimentally established.

Other mitochondria-targeted antioxidants have likewise demonstrated context-dependent protective effects. Comparative analyses by Liu et al. revealed differential antioxidative efficiencies between MitoTEMPO and SkQ1 under oxidative stress conditions, suggesting distinct capacities for modulating mtROS dynamics [94]. In renal injury models, MitoTEMPO has frequently been used to interrogate mtROS-dependent inflammatory and metabolic signaling pathways. Hou et al. demonstrated that suppression of mtROS by MitoTEMPO attenuated NLRP3 inflammasome activation, whereas Yu et al. showed that MitoTEMPO improved mitochondrial function and enhanced autophagic responses in cisplatin-treated kidneys and HK-2 cells [95,96]. Beyond direct ROS scavenging, mitochondria-targeted peptides such as SS-31 (elamipretide) exert protective effects by stabilizing cardiolipin and preserving mitochondrial cristae architecture [97]. Yang et al. reported that SS-31 alleviated cisplatin-induced acute kidney injury by suppressing mitochondrial ROS-NLRP3 inflammasome signaling [98], while Whitson et al. demonstrated its broader capacity to improve mitochondrial metabolism in aging tissues [99].

Importantly, emerging evidence suggests that mitochondria-targeted antioxidant therapies may exert context-dependent, and in some cases paradoxical, effects. Excessive suppression of mtROS may interfere with physiological redox signaling, stress adaptation, and mitophagic-mediated quality control. In addition, differential responses between animal models and human tissues remain a major translational challenge. Indeed, several studies have reported that certain mitochondria-targeted antioxidants, including MitoQ derivatives, can induce mitochondrial swelling and membrane depolarization under specific experimental conditions, suggesting the complexity of mitochondrial redox modulation [100,101]. Collectively, these findings suggest that mitochondria-directed antioxidants function not simply as ROS scavengers but as broader modulators of mitochondrial stress adaptation, inflammatory amplification, and mitochondrial quality control. However, current evidence linking these therapies to direct regulation of MAM integrity or adaptive MAM remodeling in kidney transplantation remains limited. Future studies integrating spatial analysis of ER-mitochondria communication with transplantation-relevant models, including ex vivo machine perfusion systems, will be important for defining the mechanistic specificity of MAM-targeted antioxidant strategies.

### 5.2 Modulation of ER oxidoreductases and redox homeostasis

In addition to directly targeting mtROS, modulation of ER-localized oxidoreductases has emerged as an important strategy for regulating redox signaling at ER–mitochondria interfaces. Because oxidative protein folding within the ER is tightly coupled to ROS generation and calcium signaling, dysregulation of ER redox enzymes can propagate oxidative stress and mitochondrial dysfunction during ischemic injury. Among these enzymes, endoplasmic reticulum oxidoreductin-1α (ERO1α) has attracted increasing attention as a critical mediator linking ER oxidative stress to mitochondrial injury. Hamilton et al. demonstrated that ERO1α-dependent dissociation of ERp44 from ryanodine receptors promotes aberrant calcium release and oxidative stress signaling in cardiomyocytes [102]. Similarly, Spina et al. showed that ERO1α contributes to arsenite-induced mitochondrial ROS accumulation through functional interaction with calcium signaling pathways [103]. Although these studies were not conducted in renal transplantation models, they provide important mechanistic evidence supporting the concept that ER oxidoreductases can amplify stress signaling across ER-mitochondria networks under pathological conditions. Protein disulfide isomerase (PDI) represents another redox-sensitive node involved in ER–mitochondria stress adaptation. Pokkunuri et al. reported that pharmacological inhibition of PDI in renal proximal tubular cells disrupts Keap1-NRF2 signaling, impairs mitochondrial function, and promotes apoptosis, thereby underscoring the close functional coupling between ER redox regulation and mitochondrial homeostasis [104]. These findings further suggest that ER oxidoreductases influence cellular stress responses not only through regulation of protein folding, but also through broader effects on mitochondrial quality control, antioxidant signaling, and metabolic adaptation.

In parallel, pharmacological activation of NRF2-dependent antioxidant pathways has been explored as an indirect strategy to reinforce ER and mitochondrial redox buffering capacity. Bardoxolone methyl, a potent NRF2 activator, demonstrated renoprotective and metabolic effects in experimental and clinical studies of chronic kidney disease [105]. However, despite initial enthusiasm, subsequent clinical outcomes revealed important limitations, including cardiovascular adverse events and inconsistent therapeutic efficacy, highlighting the complexity of systemic redox modulation in human disease [106]. Similarly, we previously demonstrated that methyl eugenol enhances NRF2 nuclear retention through AMPK/GSK3β signaling, thereby attenuating oxidative stress-induced renal injury [2]. Nevertheless, although NRF2 activation consistently attenuates oxidative injury in preclinical models, its translational efficacy has remained variable and may be influenced by compensatory redox pathways, off-target effects, and differences between experimental systems and clinical settings [107,108]. Collectively, these observations suggest that therapeutic modulation of ER redox homeostasis is unlikely to function through simple global suppression of ROS. Rather, ER oxidoreductases and NRF2-associated pathways appear to regulate dynamic stress adaptation across ER-mitochondria signaling networks by reshaping calcium signaling, mitochondrial function, antioxidant capacity, and cellular proteostasis. At the same time, the context-dependent and occasionally paradoxical, effects of redox-targeted interventions highlight the need for more precise therapeutic strategies capable of selectively modulating adaptive versus maladaptive stress signaling during kidney transplantation.

### 5.3 Regulation of MAM-associated signaling proteins

Beyond redox-modulating enzymes, multiple structural and signaling proteins localized at MAM critically regulate calcium transfer, mitochondrial dynamics, and stress adaptation. Emerging evidence suggests that these MAM-resident proteins function not only as structural tethers, but also as dynamic regulators of stress-responsive inter-organelle communication. As such, they may represent mechanistically more precise therapeutic targets than nonspecific antioxidant strategies. Among these regulators, MFN2 has emerged as a central mediator of ER–mitochondria tethering and stress adaptation. Song et al. demonstrated that MFN2 overexpression attenuates sorafenib-induced cardiomyocyte necroptosis through regulation of CaMKIIδ signaling at MAM interfaces [109]. Similarly, Yepuri et al. identified a DIAPH1–MFN2 interaction that modulates ER–mitochondria contacts and cellular responses to ischemic and hypoxic stress [63]. Notably, however, the precise role of MFN2 in ER-mitochondria tethering remains incompletely resolved. While some studies support a role for MFN2 in promoting organelle coupling, others suggest context-dependent regulatory, or even antagonistic, effects on ER-mitochondria proximity [110,111]. These findings highlight the complexity and dynamic nature of MAM remodeling under stress conditions. Several UPR-associated signaling molecules also exert important noncanonical functions at MAM interfaces. Lebeau et al. demonstrated that the PERK arm of the UPR regulates mitochondrial morphology during acute ER stress independently of its transcriptional activity, thereby linking ER stress sensing directly to mitochondrial remodeling [112]. Pharmacological inhibition of PERK using agents such as GSK2606414 has therefore been proposed as a potential strategy to limit ER stress-associated injury [113]. Importantly, the key supporting studies were conducted primarily in thapsigargin-induced ER stress models rather than in renal ischemia-reperfusion or transplantation systems [112]. Given that the PERK-eIF2α pathway mediates both adaptive proteostatic responses and maladaptive apoptotic signaling, therapeutic PERK inhibition is likely to exert highly context-dependent effects during renal IRI. Meanwhile, accumulating evidence supports a pivotal role for IRE1 in regulating ER-mitochondria communication beyond its canonical UPR functions. Carreras-Sureda et al. showed that IRE1 modulates ER–mitochondria coupling and calcium transfer by shaping MAM composition, thereby directly influencing bioenergetic capacity and stress adaptation [91]. In parallel, ERAD pathways have also been implicated in the regulation of mitochondrial homeostasis and stress adaptation at ER–mitochondria interfaces. Zhou et al. reported that ERAD-associated components modulate mitochondrial dynamics and metabolic function in highly active cells, suggesting that proteostasis control within the ER may directly influence mitochondrial quality control and organelle remodeling [114]. These findings further support the concept that MAM-associated signaling integrates not only calcium and redox communication but also coordinated proteostatic regulation across intracellular organelles during cellular stress. Despite extensive mechanistic evidence implicating IRE1-XBP1 signaling in renal injury, therapeutic modulation of this pathway in transplantation settings remains insufficiently explored. Agents targeting IRE1 kinase or RNase activity, including KIRA-family inhibitors, may therefore represent promising strategies for selectively attenuating maladaptive ER stress signaling while preserving adaptive proteostatic responses.

Additional regulatory complexity arises from MAM-associated scaffold and chaperone proteins that stabilize ER-mitochondria signaling complexes. Liu et al. identified DJ-1 as an important stabilizer of the IP3R–GRP75–VDAC complex, essential for maintaining ER–mitochondria integrity and mitochondrial function [115]. Extending this concept, Yuan et al. demonstrated that the IP3R–GRP75–VDAC axis mediates ER stress–induced mitochondrial oxidative injury in diabetic atrial remodeling, further supporting the pathological relevance of stress-responsive calcium transfer signaling at MAM interfaces [116]. Moreover, the Sig-1R, discussed earlier as a MAM-enriched chaperone, has also attracted increasing pharmacological interest due to its ability to stabilize IP3R signaling, regulate calcium transfer, and enhance cellular stress resilience under ischemic conditions. Together, these studies support the concept that MAM-resident signaling proteins function as dynamic regulators of stress adaptation rather than passive structural scaffolds alone. Targeting these pathways therefore offers opportunities to modulate calcium flux, redox balance, and mitochondrial resilience under stress conditions (Table 1).

**Table 1:** Comprehensive overview of therapeutic agents or targets related to MAM.

Category	Compound/inhibitor	Target/Mechanism	Disease/Model	Reference
Antioxidants	MitoQ	Selectively scavenges mtROS and limits lipid peroxidation	Renal IRI, diabetic kidney disease (DKD)	[92,93]
	SkQ1	Suppresses mtROS production	Renal IRI model	[94]
	MitoTEMPO	Reduces mitochondrial O_2_^−^ and ROS propagation	Diabetic nephropathy (DN), acute kidney injury (AKI) models	[95,96]
	SS-31	Stabilizes cristae structure and reduces ROS generation	Acute kidney injury (AKI), aged heart models	[[97], [98], [99]]
Modulation of oxidoreductases	ERO1α (e.g., genetic or EN460)	Reduces ROS production during oxidative protein folding	Model of hypertrophy; ER stress models	[102,103]
	PDI	Alters ER oxidative folding load and ER redox balance	HK2 cells	[104]
	NRF2 (e.g., bardoxolone methyl)	Activates antioxidant pathways and influences ER and mitochondrial redox buffering capacity	Diabetic kidney disease, Renal IRI model	[2,105]
MAM-signaling proteins	MFN2 (genetic or pharmacologic)	Controls ER-mitochondria tethering strength and stress signaling	Cardiomyocyte necroptosis, IRI models	[63,109]
	PERK inhibitors (e.g., GSK2606414)	Modulates PERK-dependent UPR signaling at MAM	Thapsigargin-induced ER stress model	[112]
	IRE1α	Fine-tunes ER and mitochondria communication	IRE1α knockout (KO) cells	[91]
	Sigma-1 receptor (Sig-1R)	Stabilizes IP3R–GRP75–VDAC complexes and ER–mitochondrial stress signaling	Brown adipocytes	[114]
	GRP75 modulation (genetic)	Regulates physical ER–mitochondria coupling and Ca^2+^ microdomains	Diabetic model and M17 cell lines	[115,116]

Collectively, emerging evidence support the concept that MAM function as actionable signaling hubs that coordinate oxidative stress, ER stress, and mitochondrial dysfunction in kidney transplantation. Although the above-mentioned direct clinical targeting of MAM remains in its early stages, preclinical studies have already demonstrated that modulation of MAM-associated signaling can restore ER–mitochondria communication, reduce oxidative stress, and confer functional protection in experimental models. Nevertheless, in many cases, mechanistic links between therapeutic benefit and direct preservation of MAM integrity remain inferred rather than conclusively demonstrated. Importantly, the effects of MAM-targeted interventions are likely to be highly context- and time-dependent. Interventions administered during ischemic preconditioning, organ preservation, or post-transplant reperfusion may differentially influence distinct phases of stress adaptation and injury progression. In this regard, ex vivo machine perfusion has emerged as a particularly promising platform for targeted drug delivery and mechanistic intervention, enabling modulation of oxidative and metabolic injury pathways in donor kidneys prior to implantation [[117], [118], [119]]. Such approaches may provide unique opportunities to selectively enhance adaptive stress responses while limiting maladaptive injury amplification during transplantation. Future progress will rely on integrating detailed molecular dissection with advanced human-relevant platforms to refine target specificity, define therapeutic windows, and minimize unintended interference with physiological stress responses. Ultimately, a deeper understanding of dynamic ER–mitochondria communication may facilitate the development of precision therapeutic strategies aimed at preserving mitochondrial and ER homeostasis, thereby enhancing graft resilience and improving long-term transplant outcomes.

## 6 Organoids: new platforms

Conventional 2D cell culture models and animal systems incompletely recapitulate human-specific redox regulation, stress sensitivity, and aging-related susceptibility, whereas human biopsy material offers only static snapshots of injury without experimental manipulability [120]. These limitations highlight the need for complementary human-relevant platforms that enable dynamic and mechanistic interrogation of stress signaling pathways. In this context, human organoids have emerged as a versatile experimental system that preserves cell-type composition, tissue architecture, and inter-organelle communication, thereby providing a physiologically relevant platform for studying MAM-dependent signaling. Importantly, organoids enable controlled perturbation of redox balance and ER stress while preserving functional ER-mitochondria crosstalk [121], making them particularly well-suited for dissecting how MAM integrity governs cellular stress adaptation and injury responses. Beyond their mechanistic value, organoids also serve as translational platforms for functional phenotyping, therapeutic screening, and precision stratification in transplantation medicine [122,123]. By enabling simultaneous assessment of mitochondrial function, ER stress responses, and inter-organelle communication, organoid systems provide a unique bridge between molecular mechanisms and clinically relevant phenotypes. Recent work from our group has contributed to the development of microengineered 3D organoid platforms that enable controlled and reproducible modeling of epithelial stress responses under dynamic conditions [124]. Using pump-free or microfluidic-based organoid systems, we have demonstrated the feasibility of maintaining stable perfusion, preserving tissue architecture, and applying defined chemical or environmental stimuli to investigate stress-related cellular responses. Thereby further providing a methodological foundation for extending organoid-based approaches to study MAM-dependent ER–mitochondria communication in kidney transplantation research under physiologically relevant conditions [125]. Collectively, these features position organoids not merely as structural tissue surrogates, but as functional platforms for interrogating MAM-centered stress signaling networks, thereby bridging reductionist in vitro systems and complex in vivo models.

### 6.1 Kidney organoids

Kidney organoids derived from human pluripotent cells (PSC) or adult stem cells (ASC) have been previously described as important human-relevant platforms in detail [126,127]. These systems partially recapitulate key architectural, cellular, and transcriptional features of developing renal tissue while retaining substantial experimental accessibility. Their 3D organization of kidney organoids enables more physiologically relevant cell–cell interactions, metabolic coupling, and spatial stress signaling, thereby providing a more suitable framework for studying oxidative injury and ER stress responses in renal epithelial cells. Importantly, organoids permit precise control over environmental variables such as oxygen tension, nutrient availability, inflammatory stimuli, and pharmacologic perturbations, making them particularly suited for dissecting dynamic stress responses [11]. In this context, organoids enable direct mechanistic interrogation of processes that are difficult to resolve in vivo, including early redox signaling events, temporal activation of UPR phases, and spatial coordination between mitochondria and the ER. Their compatibility with live-cell imaging, CRISPR-based genetic manipulation, transcriptomic profiling, and spatial multi-omics further enhances their value for mechanistic investigation. For translational research, organoids occupy a unique intermediate position between reductionist in vitro systems and whole-organ transplantation models. They provide a human-relevant experimental platform in which mechanistic hypotheses can be tested under defined conditions while retaining sufficient biological complexity to inform clinical relevance. Several organoid platforms described in the following sections have been developed, each offering complementary advantages for studying redox and ER stress mechanisms.

### 6.2 PSC-derived kidney organoids

PSC-derived kidney organoids remain the most widely used human renal organoid systems for studying developmental biology, kidney injury, and stress signaling pathways. Generated either from embryonic stem cells (ESC) or PSC following induction by stepwise differentiation protocols that recapitulate nephrogenesis (iPSC), these organoids consist of multiple nephron-like segments and stromal populations [128]. Their developmental plasticity, scalability, and compatibility with genetic engineering make PSC-derived kidney organoids particularly suitable for mechanistic interrogation of oxidative stress, ER stress, mitochondrial dysfunction, and hypoxia-reoxygenation injury. In addition, these systems are highly amenable to live imaging, CRISPR-based perturbation, and multi-omics analyses, allowing dynamic evaluation of stress-responsive signaling pathways across defined differentiation stages. Despite these advantages, current PSC-derived kidney organoids retain substantial limitations. Most notably, proximal tubular compartments remain incompletely matured and exhibit reduced mitochondrial density, immature transporter expression, altered metabolic programming, and limited oxidative phosphorylation capacity relative to adult proximal tubules [[129], [130], [131]]. These deficiencies are particularly relevant because proximal tubular epithelial cells are among the most metabolically active cell types in the kidney and are highly susceptible to redox imbalance. In addition, PSC-derived organoids generally lack functional vascularization, immune cell integration, and physiological oxygen gradients, all of which critically influence oxidative injury and inflammatory stress amplification during transplantation. In summary, although their fetal-like immaturity remains a limitation and may not fully recapitulate the bioenergetic vulnerability and redox dynamics of mature transplanted kidneys, they nonetheless provide valuable mechanistic insight into stress-responsive signaling pathways.

### 6.3 Proximal tubule (PT)-enriched organoids

To address some of the metabolic and functional limitations of conventional PSC-derived organoids, proximal tubule (PT)-enriched organoid systems have recently been developed as more specialized platforms for studying tubular injury and stress adaptation [130,132]. By biasing differentiation toward PT lineages, these organoids exhibit enhanced expression of transporter proteins, increased mitochondrial content, greater oxidative metabolism, and improved sensitivity to toxic and ischemic injury stimuli [133]. Given that PT epithelial cells are primary targets of IRI during kidney transplantation, PT-enriched organoids are particularly well suited for investigating mitochondrial dysfunction, ROS accumulation, ER stress activation, ferroptosis susceptibility, and metabolic maladaptation under stress conditions. Their more mature bioenergetic phenotype may therefore provide a more physiologically relevant framework for studying redox-sensitive injury pathways than conventional PSC-derived organoids. Nevertheless, direct investigation of ER-mitochondria contact dynamics, MAM integrity, or MAM-dependent calcium transfer in PT-enriched organoids has yet to be comprehensively established. Despite these limitations, PT-enriched organoids represent an important advance toward metabolically relevant human kidney injury models and may prove particularly valuable for investigating adaptive versus maladaptive stress responses in highly oxidative tubular epithelial cells.

### 6.4 Adult stem cell-derived tubuloids

Adult stem cell-derived tubuloids provide a complementary platform with distinct translational advantages. Generated from patient kidney tissue or urinary epithelial cells, these systems retain donor-specific genetic, epigenetic, and metabolic characteristics while exhibiting relatively mature epithelial phenotypes together with robust long-term expansion capacity [134,135]. Compared with PSC-derived organoids, tubuloids often exhibit more differentiated tubular features and may therefore more accurately reflect patient-specific stress susceptibility and therapeutic responsiveness. Their patient specificity makes tubuloids particularly attractive for investigating inter-individual variability in stress susceptibility and therapeutic responses, which is a critical consideration for personalized transplantation strategies. However, despite the increasing use of tubuloids to model hypoxia-reoxygenation injury and oxidative stress responses, direct investigation of MAM integrity, ER-mitochondria contact dynamics, or MAM-dependent calcium transfer in these systems remains extremely limited. Existing studies primarily assess global oxidative stress phenotypes, mitochondrial dysfunction, or ER stress activation, whereas mechanistic involvement of MAM signaling pathways is generally inferred rather than experimentally demonstrated [136]. Furthermore, technical variability across donor sources and culture conditions continues to affect reproducibility and broader translational applicability. Collectively, PSC-derived organoids, PT-enriched organoids, and adult stem cell-derived tubuloids should therefore be viewed as complementary rather than competing experimental systems. Each platform captures distinct aspects of renal stress biology while differing in developmental maturity, metabolic phenotype, structural complexity, and translational relevance. Although organoid-based systems provide valuable opportunities to study oxidative injury and stress adaptation in human-relevant settings, their ability to faithfully model dynamic MAM remodeling and ER-mitochondria crosstalk during kidney transplantation remains to be fully established.

### 6.5 Kidney organoid-based models for redox-ER stress signaling

Kidney organoids have been increasingly utilized to model oxidative injury and stress-responsive signaling through controlled exposure to diverse stressors that partially recapitulate clinically relevant renal insults [137]. Hypoxia-reoxygenation paradigms are commonly used to simulate ischemia-reperfusion-like conditions, inducing ROS accumulation, mitochondrial dysfunction, and cellular injury patterns reminiscent of transplant-related damage [138]. Because these systems permit precise temporal control over oxygen deprivation and recovery phases, they provide valuable opportunities to dissect early redox signaling events and stress adaptation dynamics in human renal cells. Inflammatory stimuli such as lipopolysaccharide (LPS) have also been applied to kidney organoids to reproduce inflammation-associated oxidative stress and epithelial [139]. LPS exposure activates innate immune signaling pathways and promotes ROS generation, mitochondrial depolarization, and cell death, thereby enabling the study of epithelial-intrinsic inflammatory responses in the absence of systemic immune confounders. Additional injury paradigms include exposure to hemin or other pro-oxidant compounds, which induce robust oxidative stress and mitochondrial injury [140]. These models are particularly relevant for studying hemolysis-associated or toxin-mediated renal damage. Importantly, the integration of fluorescent stress reporters and live-cell imaging approaches has substantially expanded the analytical capabilities of organoid systems. Organoids engineered with HMOX1-based oxidative stress sensors, mitochondrial membrane potential reporters, or calcium-sensitive probes enable real-time visualization and quantitative analysis of cellular stress responses at high spatial resolution [141]. Transcriptomic and proteomic analyses of treated organoids provide a powerful means to capture human-relevant molecular programs associated with redox-ER stress and stress-responsive transcriptional networks. A major advantage of organoid systems is their ability to preserve spatial epithelial organization, thereby allowing analysis of cell type-specific stress vulnerability within structured nephron-like compartments. Coupled with CRISPR/Cas-based perturbation strategies, these platforms provide increasingly powerful tools for mechanistic interrogation of stress-responsive signaling networks in human renal cells [142]. Although direct experimental studies assessing MAM dynamics or MAM-dependent calcium transfer remain limited, kidney organoids offer strong conceptual and technical potential for modeling ER-mitochondria communication. In particular, emerging organoid-on-chip platforms incorporating perfusion and mechanical cues further enhance physiological relevance by improving oxygen delivery and metabolic coupling [143]. Such systems may enable more faithful modeling of ischemia-reperfusion-induced calcium overload, ROS amplification, and mitochondrial dysfunction. Collectively, kidney organoids provide valuable human-relevant platforms for studying oxidative injury and ER stress adaptation under controlled conditions; however, their capacity to faithfully model dynamic MAM remodeling and transplantation-specific inter-organelle crosstalk remains incompletely established. Future integration of vascularization strategies, immune components, advanced imaging technologies, and organoid-on-chip systems will likely be essential for advancing these systems toward more physiologically relevant models of redox-sensitive ER-mitochondria communication in kidney transplantation.

### 6.6 Translational implications and current limitations

**Fig. 5:**
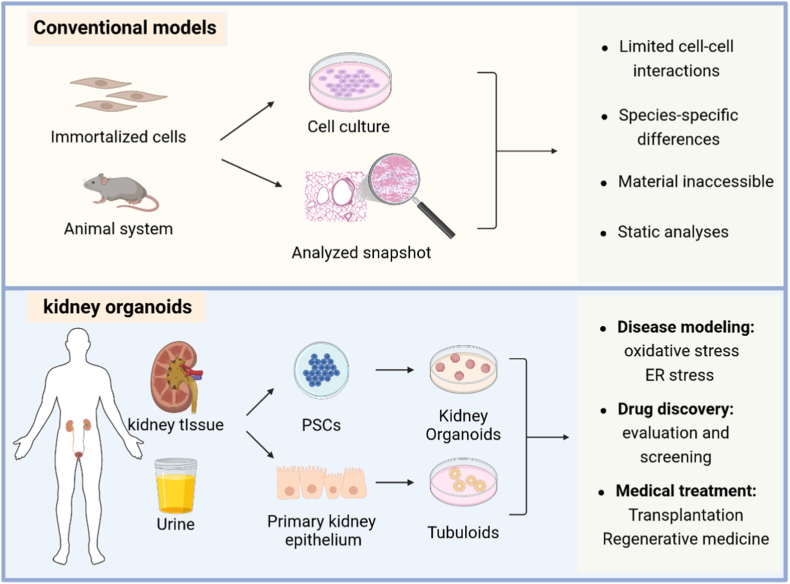
Schematic illustration of conventional experimental models and kidney organoid-based platforms for studying kidney injury and stress responses. The upper panel illustrates conventional experimental models, including immortalized cell cultures and animal systems, which are widely used to study kidney injury but are limited by simplified cell-cell interactions, species-specific differences, restricted accessibility to human tissue, and predominantly static endpoint analyses. In contrast, kidney organoid-based approaches leverage human kidney tissue, urine samples, pluripotent stem cells (PSCs), or primary renal epithelial cells. These systems enable the generation of kidney organoids or tubuloids that more closely recapitulate human renal architecture and cellular heterogeneity. Kidney organoids provide versatile platforms for modeling disease-relevant stress conditions, such as oxidative stress and endoplasmic reticulum (ER) stress, for drug evaluation and screening, and for exploring translational applications including transplantation and regenerative medicine. Created with BioRender.com.

Taken together, kidney organoids represent increasingly sophisticated platforms for investigating oxidative stress, ER stress, mitochondrial dysfunction, and stress-responsive signaling in renal epithelial cells. Their experimental accessibility, compatibility with live imaging and genetic manipulation, and ability to preserve 3D tissue organization provide important advantages for dissecting complex stress networks under controlled conditions. By enabling controlled perturbation of redox balance, organoid systems bridge a critical gap between molecular biology and translational transplantation research. Nevertheless, incomplete vascularization, the absence of immune and stromal components, immature metabolic phenotypes, and limited modeling of physiologic oxygen gradients constrain the ability of current organoid systems to fully recapitulate the complexity of renal IRI. Importantly, direct experimental interrogation of MAM integrity and MAM-dependent calcium signaling in kidney organoids remains largely absent. Thus, current evidence linking organoid-associated stress phenotypes to MAM remodeling remains predominantly inferential. Despite these limitations, ongoing advances in vascularized organoids, organoid-on-chip systems, spatial multi-omics, and high-resolution imaging technologies are expected to substantially improve the physiologic relevance and mechanistic resolution of these systems [144]. As these technologies continue to evolve, they may provide increasingly powerful platforms for investigating adaptive versus maladaptive MAM remodeling, identifying stress-responsive therapeutic targets, and facilitating translational studies of kidney transplantation injury, opening new avenues for regenerative approaches to renal replacement and repair [145,146] (Fig. 5).

## 7 Conclusions and future perspectives

**Fig. 6:**
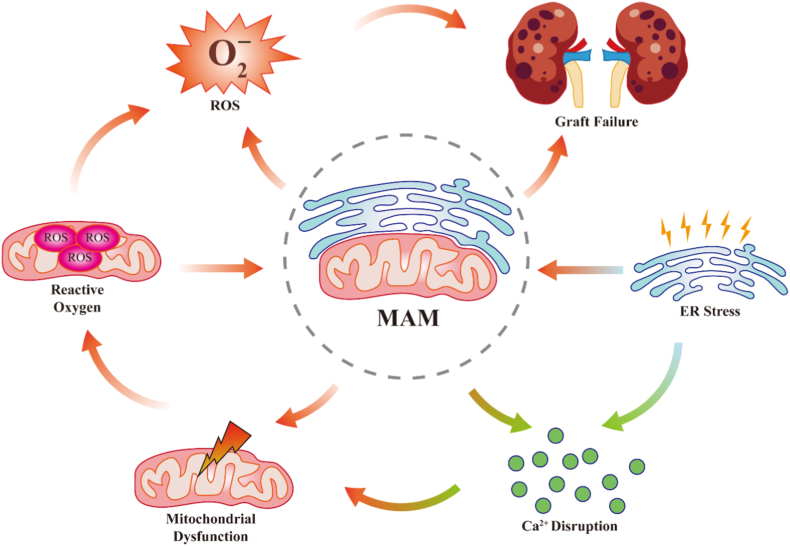
Graphical illustration of the central role of MAM. Mitochondria-associated membranes (MAM) integrate mitochondrial ROS signaling and ER stress responses during renal ischemia-reperfusion injury. Redox imbalance at MAM interfaces promotes Ca^2+^ dysregulation, mitochondrial dysfunction, and persistent oxidative stress, forming a self-amplifying pathogenic circuit that accelerates graft damage. Targeting MAM-centered redox signaling networks represents a promising strategy to improve transplant outcomes.

Oxidative stress and ER stress are increasingly recognized as tightly interconnected and mutually reinforcing drivers of renal IRI and graft dysfunction during kidney transplantation. Rather than functioning as isolated pathways, these stress responses converge through dynamic ER-mitochondria communication networks that coordinate calcium signaling, redox homeostasis, proteostasis, mitochondrial quality control, and inflammatory amplification. Accumulating evidence therefore positions MAMs as central signaling platforms governing cellular adaptation and injury progression under transplantation-related stress conditions (Fig. 6). Importantly, emerging evidence suggests that MAM remodeling is highly dynamic and context-dependent. Under transient or moderate stress, adaptive MAM remodeling may support mitochondrial bioenergetics, coordinate stress signaling, and restore cellular homeostasis. In contrast, persistent oxidative stress, calcium overload, lipid peroxidation, and maladaptive UPR activation drive pathological MAM remodeling, thereby amplifying mitochondrial dysfunction, ferroptosis, inflammation, and graft injury. Defining the molecular determinants that govern this adaptive-to-maladaptive transition therefore remains a major unresolved challenge in the field.

Despite substantial mechanistic progress, several barriers continue to limit translational advancement. Much of the current evidence derives from experimental rather than human transplantation models, and direct assessment of MAM integrity, calcium transfer dynamics, and lipid remodeling in clinically relevant settings remains limited. In addition, therapeutic strategies targeting ROS, ER stress, or MAM-associated signaling frequently exhibit context-dependent effects, reflecting the dual roles of redox signaling and UPR pathways in both cellular adaptation and injury progression. These complexities highlight the need for more precise therapeutic approaches capable of selectively attenuating maladaptive stress amplification while preserving physiological stress adaptation. In addition, kidney organoids and organoid-on-chip systems represent promising human-relevant platforms for investigating stress-responsive signaling under controlled conditions. Although current models incompletely recapitulate the vascular, immune, and metabolic features of transplanted kidneys, continued advances in tissue engineering, spatial multi-omics, and live-cell imaging are expected to enable increasingly sophisticated interrogation of ER-mitochondria crosstalk and dynamic MAM remodeling in human renal tissue.

Collectively, this review positions MAMs not merely as structural contact sites, but as dynamic signaling hubs integrating oxidative stress, ER stress, mitochondrial dysfunction, and intracellular organelle communication during kidney transplantation. A deeper understanding of stress-responsive MAM remodeling, together with continued development of kidney organoid-based models, may ultimately facilitate the development of more precise therapeutic strategies aimed at preserving mitochondrial and ER homeostasis, improving graft resilience, and enhancing long-term transplant outcomes.

## Ethics approval and consent to participate

Not applicable.

## Consent for publication

All authors have read and approved the final manuscript and agree to its publication.

## CRediT authorship contribution statement

**Baicheng Kuang:** Conceptualization, Validation, Writing – original draft. **Lin Han:** Conceptualization, Investigation, Writing – original draft. **Sopheaktra Tan:** Conceptualization, Software, Visualization. **Sokun Tan:** Supervision, Visualization. **Sopheap Bou:** Methodology, Validation. **Yuanyuan Zhao:** Investigation, Software. **Yan Li:** Methodology, Supervision, Visualization. **Jiasheng Yu:** Validation, Writing – review & editing. **Nianqiao Gong:** Conceptualization, Funding acquisition, Validation, Writing – review & editing.

## Declaration of competing interest

The authors declare the following financial interests/personal relationships which may be considered as potential competing interests:Reports a relationship with that includes:. Has patent pending to. If there are other authors, they declare that they have no known competing financial interests or personal relationships that could have appeared to influence the work reported in this paper.

## Data Availability

No data was used for the research described in the article.

## Abbreviations:

ATF4: activating transcription factor 4
ATF6: activating transcription factor 6
ATG14: autophagy-related protein 14
cGAS: cyclic GMP–AMP synthase
DRP1: dynamin-related protein 1
ΔΨm: mitochondrial membrane potential
eIF2α: eukaryotic initiation factor 2 alpha
ER: endoplasmic reticulum
FUNDC1: FUN14 domain-containing protein 1
GRP75: glucose-regulated protein 75
IP3R: inositol 1,4,5-trisphosphate receptor
IRE1: inositol-requiring enzyme 1
IRI: ischemia-reperfusion injury
MAM: mitochondria-associated membranes
MCU: mitochondrial calcium uniporter
MFN1/2: mitofusin 1/2
mPTP: mitochondrial permeability transition pore
mtDNA: mitochondrial DNA
Mφ: macrophage
NOX: NADPH oxidases
NOS: nitric oxide synthase
PINK1: PTEN-induced kinase 1
PERK: protein kinase RNA-like ER kinase
ROS: reactive oxygen species
Sig-1R: sigma-1 receptor
STING: stimulator of interferon genes
TLR: Toll-like receptor
UPR: unfolded protein response
VDAC: voltage-dependent anion channel
XBP1: X-box binding protein 1
XBP1s: spliced X-box binding protein 1
XOR: xanthine oxidoreductase
